# Prior knowledge-guided multilevel graph neural network for tumor risk prediction and interpretation via multi-omics data integration

**DOI:** 10.1093/bib/bbae184

**Published:** 2024-04-25

**Authors:** Hongxi Yan, Dawei Weng, Dongguo Li, Yu Gu, Wenji Ma, Qingjie Liu

**Affiliations:** Department of Computer Science, Beihang University, XueYuan Road, 100191, BeiJing, China; School of Biomedical Engineering, Capital Medical University, 10 You An Men WaiXi Tou Tiao, 100069, Beijing, China; School of Biomedical Engineering, Capital Medical University, 10 You An Men WaiXi Tou Tiao, 100069, Beijing, China; School of Biomedical Engineering, Capital Medical University, 10 You An Men WaiXi Tou Tiao, 100069, Beijing, China; Center for Single-Cell Omics, School of Public Health, Shanghai Jiao Tong University School of Medicine, 227 South Chongqing Road, 200025, Shanghai, China; Department of Computer Science, Beihang University, XueYuan Road, 100191, BeiJing, China

**Keywords:** graph neural network, multi-omics, pathway, risk classification, interpretability

## Abstract

The interrelation and complementary nature of multi-omics data can provide valuable insights into the intricate molecular mechanisms underlying diseases. However, challenges such as limited sample size, high data dimensionality and differences in omics modalities pose significant obstacles to fully harnessing the potential of these data. The prior knowledge such as gene regulatory network and pathway information harbors useful gene–gene interaction and gene functional module information. To effectively integrate multi-omics data and make full use of the prior knowledge, here, we propose a Multilevel-graph neural network (GNN): a hierarchically designed deep learning algorithm that sequentially leverages multi-omics data, gene regulatory networks and pathway information to extract features and enhance accuracy in predicting survival risk. Our method achieved better accuracy compared with existing methods. Furthermore, key factors nonlinearly associated with the tumor pathogenesis are prioritized by employing two interpretation algorithms (i.e. GNN-Explainer and IGscore) for neural networks, at gene and pathway level, respectively. The top genes and pathways exhibit strong associations with disease in survival analyses, many of which such as SEC61G and CYP27B1 are previously reported in the literature.

## INTRODUCTION

To achieve a comprehensive and multidimensional understanding of complex and systemic diseases such as cancer, it is crucial to integrate multiple types of omics data [1–3], as analyzing a single omics offers only a partial understanding. While omics data can reveal various aspects of the human genome and enhance our understanding of cancer biology, the limited sample size available poses a significant challenge for the application of machine learning and deep learning techniques.

In recent years, deep learning tools have become increasingly prominent in oncology. Deep learning models have been extensively used for various tasks, including survival prediction [4–6], regulatory process analysis [7, 8], cancer classification [9–11], unveiling molecular mechanisms [12], identifying drug–target interactions [13] and yielding remarkable results. However, the high cost of acquiring multi-omics data for each patient often results in specific cancer-related multi-omics datasets having relatively small sample sizes. This can lead to overfitting and the curse of dimensionality when applying machine learning and deep learning methods to multi-omics data. Additionally, multi-omics data itself are plagued by issues such as high noise levels, high missing rates and batch effects that severely impede the accuracy of model predictions. To effectively address these issues, numerous existing methodologies incorporate prior knowledge and network knowledge into their frameworks to alleviate the difficulty of model training while enhancing both generalization capabilities and interpretability [14].

Pathway information and regulatory network information are the most commonly used prior knowledge in recent studies. Gene pathways can integrate molecular information, covering physiological processes such as metabolism, cellular processes and human diseases, making them widely employed in omics data analysis. Pathway information can be obtained from the extensively used KEGG database [15], which provides gene pathway details and functional annotations. Many methods leverage gene pathways to guide network structure design or feature extraction algorithms. Elmarakeby *et al.* [16] developed a fully interpretable network that sequentially extracts gene, pathway and biological process features to predict disease states. DeepOmix [4] aggregates multi-omics data based on pathways and uses fully connected layers for prediction. PathCNN [17] compresses multi-omics data based on gene pathways using PCA. The compressed multi-omics features are then combined into an omics image that is subsequently fed into a Convolution Neural Network for risk prediction. This method employs Grad-CAM [18] to explain the prediction results and identify differential pathways for long-term and non-long-term survival patients. However, these methods often rely solely on pathway information and use simple linear aggregation to combine omics data into pathway information, limiting their ability to provide comprehensive insights. Incorporating a regulatory network can supplement more detailed information for the neural network, enhancing its effectiveness.

The previous approaches [19, 20] to studying biological processes are based on molecular interaction networks between individual biological molecules, where nodes represent biological molecules, and edges describe the interactions between pairs of nodes. Multiple types of biological interaction networks representing different biological mechanisms [21] exist, based on various types of interactions such as protein–protein interaction networks [22] and gene regulatory networks [23]. Graph neural networks (GNNs) are commonly used for analyzing graph data in cancer research, where interaction networks provide supplementary prior knowledge. GNNs have been employed for tasks such as inferring regulatory networks [24], metastatic classification [25], cancer type classification [26], key gene prediction [27] and survival prediction [28–30]. However, few works have considered how to better utilize regulatory network knowledge across multiple omics datasets. The multilevel GNN employs a guidance graph that encompasses inter-omics and intra-omics regulatory relationships, thereby facilitating a more effective adaptation to multiomics data.

In this study, a new approach has been proposed to predict the risk of survival of patients. We propose a hierarchical deep learning model approach that comprises three components: a gene encoding graph neural network, a pathway aggregation block and a prediction module, corresponding to the extraction of gene information, pathway information and patient risk information, respectively. First, construct a guidance graph to establish regulatory relationships between genes and connections between different omics datasets. Gene encoding graph neural network generates gene-level features through a graph neural network guided by a guidance graph. Based on pathway information, the gene-level features are aggregated into pathway-level features in the pathway aggregation block through a Principal Component Analysis (PCA) initialized learnable layer, and further feature extraction is performed through a few hidden layer. The prediction module utilizes high-level pathway-level features to predict patient risk with pooling and fully connected (FC) layers. The multilevel graph neural network conducts further compression and extraction of features across various levels. At the gene level, it utilizes mutual information to filter out features of limited informational value, reducing superfluous data. Moreover, it leverages regulatory networks to explicitly guide the neural network in extracting intra-omics and inter-omics information, structurally avoiding the fusion of irrelevant gene information, thus enhancing the quality of gene-level features. At the pathway level, the multilevel graph neural network employs gene pathway information to further compress and extract gene features that collaboratively fulfill biological functions, reducing redundant information and increasing feature discrimination. Through such multi-level information extraction and compression, the multilevel graph neural network is able to unearth more valuable insights, consequently achieving superior performance on small datasets. The multi-level design of the model also bring improved interpretability, allowing for analysis of key factors from multiple dimensions. The study uses a node-mask-based explanation method to identify crucial genes and the Integrated Gradients method [31] to identify critical gene pathways. Many of these crucial genes and pathways have been confirmed in past studies. Survival analysis of the identified crucial genes and pathways was conducted. These crucial genes and pathways have a strong correlation with patient risk, demonstrating the significance of the model’s explanation methods.

## METHOD

### Overview

**Figure 1:**
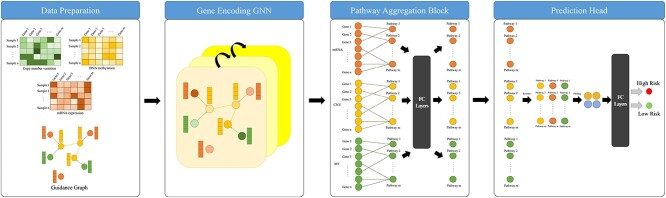
The algorithm comprises three modules: the graph neural network utilizes a guidance graph to encode features at the gene level, the pathway aggregation block aggregates gene features into pathway features based on pathway information and, finally, the prediction module uses pooling, FC layers to predict the risk.

**Figure 2:**
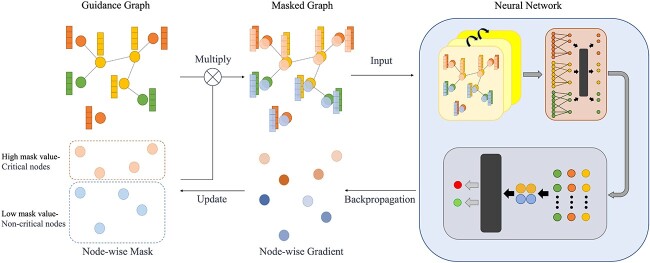
The gene interpretation algorithm assigns a mask to each node. The masked graph is obtained by multiplying the mask with the Guidance graph, and this masked graph is then input into the neural network. Through multiple iterations using gradient descent, the mask values are adjusted to minimize the error between the predicted result and the true value. In the end, critical nodes with high mask values and non-critical nodes with low mask values are obtained.

Our method takes in mRNA expression, copy number variation (CNV), DNA methylation profiling data and age as inputs, and outputs a two-dimensional (2D) vector that denotes the probabilities of low risk and high risk for each patient. For preprocessing of the data, given the low information density inherent in the omics data, mutual information on the training set was employed for gene selection for each omic data set separately. The overall framework of multilevel GNN is illustrated in Figure 1. The multilevel GNN comprises three components: a gene-encoding graph neural network that leverages both gene regulatory network and multi-omics data, aiming to enrich gene-level features with information on gene interactions based on specific regulatory relationships; a pathway aggregation block that aggregates genes within a pathway with a learnable linear layer to generate pathway-level features; and a prediction module that generates the prediction results, i.e. the probability of high and low risk of mortality, respectively. To facilitate the model with the ability of result interpretation, a mask-based neural network explanation method is employed to extract key genes that provide insights into the risk prediction. The overall structure of the key gene interpretation method is shown Figure 2. Specifically, this method utilizes gradient descent to adjust node-wise masks to minimize the conditional entropy between the output and ground truth, and the final node mask values represent the importance of the nodes. These pivotal genes are important for predicting the prognosis of patients. In addition, key pathways are identified using IGscore recognition. The IGscore, representing feature attributions, is calculated using the Integrated Gradients method, which integrates the gradients of a model’s output with respect to pathway features along a path from a baseline to the actual pathway features.

### Data preprocessing

When dealing with practical problems involving higher dimensional data, it is crucial to have a substantial amount of data for fitting in order to avoid the curse of dimensionality. Predicting gene-disease risk becomes particularly challenging due to the small number of patients for each cancer type, coupled with the high dimensionality of the genetic data. To tackle this challenge, mutual information was utilized to filter the multi-omics features. The K-nearest neighbors algorithm [32] was employed to calculate mutual information for the three omics datasets. As gene expression and methylation data are continuous, the formula for calculating mutual information is as follows: 


(1) \begin{align*} MI(x)=\psi(N)-\langle\psi(N_{x})\rangle+\psi(k)-\langle\psi(m)\rangle,\end{align*}

where $\psi (\cdot )$ denotes the digamma function, $\langle \cdot \rangle $ denotes the average function. $N_{x}$ represents the total number of data points that belong to the same class as $x$ and $m$ represents the total number of data points within the neighborhood.

In each cross-validation iteration, we calculated the mutual information between all omics data and labels based on the training set. Data with mutual information greater than the mean mutual information were retained for training.

### Guidance graph construction

To capture the connections between genes, as well as between different omics data for the same gene, we employed the same guidance graph $\mathbf{G}=(V,E)$ as GLUE [7].

Firstly, for the mRNA data, a gene regulatory network $\mathbf{G}_{mRNA}=(\mathbf{V}_{mRNA}, \mathbf{E}_{mRNA})$ using SCENIC [23] was constructed, where each edge $e_{ij}$ represents a transcription factor (TF)-target pair from gene $i$ to gene $j$. Since the SCENIC importance scores $S_{ij}$ often have values greater than 1, the scores were normalized on all edges of each TF to the range of 0 to 1. These normalized scores are then used as the weights for the mRNA gene regulatory network, denoted as $W_{mRNA}$.

To link the data from different omics together, we created edges connecting CNV-mRNA ($E_{CM}$) and methylation-mRNA ($E_{MM}$) for the same gene. Since CNV often exhibits the same trend as gene expression, we constructed a positive edge ($w_{ij}=1$) between CNV and mRNA. Furthermore, as methylation typically inhibits gene expression, we constructed a negative edge ($w_{ij}=-1$) between methylation and mRNA. The absolute values of edge weights between different omics were set to 1. 


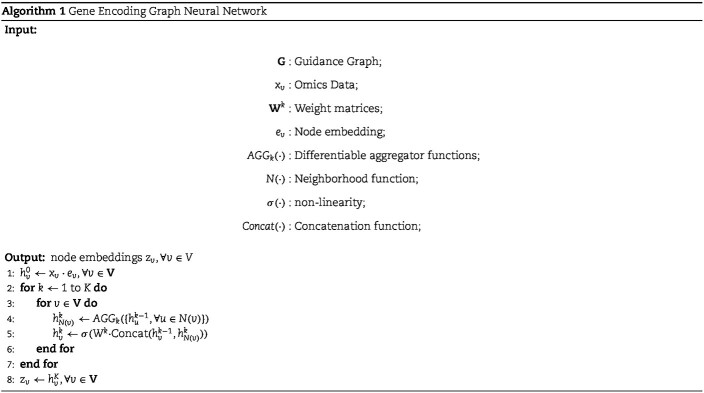


The nodes of the guidance graph consist of all nodes from each omics datasets, and the edges represent the union of edges connecting the mRNA regulatory network and different omics datasets. 


\begin{align*} &V=\{V_{mRNA}, V_{CNV}, V_{MT}\}\end{align*}

\begin{align*} &E=\{E_{mRNA}, E_{CM}, E_{MM}\}.\end{align*}

### Multilevel GNN

Multilevel GNN consists of three parts: gene encoding graph neural network, pathway aggregation block and the prediction module. Figure 1 illustrates the overall algorithm.

#### Gene encoding GNN

Defining $x_{i}$ as the expression value of the $i$th node. The gene encoding graph neural network initialize a random D-dimensional embedding $\mathbf{e}_{i}$ for each node. When a sample is fed into the neural network, the corresponding expression values are multiplied with the embeddings to obtain the input for the graph neural network $S=\{s_{v}=x_{v}\cdot e_{v},\ \forall v \in V\}$.

The gene encoding graph neural network utilizes two layers of GraphSAGE [33] to encode the omics embeddings based on the guidance graph. GraphSAGE leveraging the guidance graph to aggregate node features as follows. Firstly, embeddings are collected from the neighboring nodes of each target node within the guidance graph. Subsequently, these neighbor features are combined using a learnable aggregation function. Finally, the aggregated features are transformed through a neural network layer, resulting in an updated representation of the node. This aggregation process utilizes gene interaction information based on specific regulatory relationships to enrich gene-level features. Algorithm 1 describes the embedding generation process when considering the entire guidance graph, $\mathbf{G} = (\mathbf{V}, \mathbf{E})$, and the expression values of all nodes $X$ as input. We denote the output node embeddings as $z_{i}$ for node $i$.

#### Pathway aggregation block

After obtaining the aggregated embedding of each gene, genes within the same pathway are aggregated to obtain pathway-level features in the pathway aggregation block. Specifically, the pathway aggregation block aggregates encodings $z_{v}, \forall v \in (pathway_{i}, omic_{j})$ of nodes in the same omics within the same pathway using a learnable aggregation matrix $\mathbf{W}^{ij}_{p}$ to obtain pathway-level features, denoted as PF. To better capture pathway-level features, two FC layers are further used to encode the pathway-level features PF, resulting in the final $D$-dimensional pathway features $p_{ij}$.

For each pathway on each omics, PCA can be used to reduce N gene features to M dimensions. 


(2) \begin{align*} p^{origin}_{ij} = P_{ij}X_{ij}.\end{align*}

The matrix $\mathbf{W}^{ij}_{p}$ is initialized using the compression matrix $P_{ij}$ obtained from PCA. This initialization serves two purposes: firstly, PCA effectively compresses the features, and secondly, using a fixed initialization method improves training stability.

In addition, to maintain the independence of pathway features in each dimension, a cosine loss is applied to constrain the aggregation matrix. This constraint aims to maintain orthogonality across dimensions as much as possible. 


(3) \begin{align*} L_{ind}=\sum_{mn}\cos (W^{ijm}_{p}, W^{ijn}_{p}).\end{align*}

To emphasize the impact of abnormal genes and highlight the influence of the original omics data, the gene encoding is multiplied once again by the input omics data before performing the aggregation. The specific aggregation formula is as follows: 


(4) \begin{align*} PF_{ijm}=\mathbf{W}_{p}^{ijm}X_{v}\cdot Z_{v}.\end{align*}

The algorithm procedure is shown in Algorithm 2. 


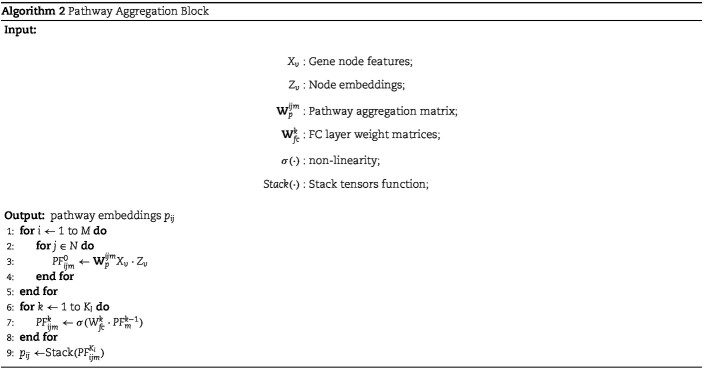


#### Prediction head

Lastly, a prediction module utilizes the pathway embeddings to predict the risk level of patients. Inspired by PathCNN, we performed sorting on the pathways before pooling. First, the pathway embeddings is arranged into a matrix $M_{p} \in R^{DNM}$, where $N$ represents the number of pathways, $M$ represents the number of multi-omic features and $D$ represents the dimension of each multi-omic feature.

The pathways are organized in the order of their pairwise Pearson similarity scores after dimensionality reduction using PCA. First, the pairwise Pearson correlation is calculated between pathways by using the original PCA features $p^{\text{origin}}_{ij}$ of each omics dataset for all samples within each pathway. The two pathways with the highest similarity score are placed at the first and second positions, followed by selecting the pathway with the highest similarity to the previous one in a sequential manner.

Due to the high dimensionality of pathway embeddings, using a FC layer directly for prediction poses significant challenges. Therefore, max pooling is applied to reduce the dimensionality of reordered pathway embeddings. At last, two linear layers are utilized to predict the final outcome.

### Gene explanation

We use a modified GNN-Explainer [35] to interpret the key genes. This demonstrates which genes are important in our prediction and, to some extent, also reflects which genes are important for the disease condition of patients.

The objective of GNN-Explainer is to select a subgraph that minimizes the conditional entropy H. 


(5) \begin{align*} H(Y|G_{S},X_{S}) = -\mathbb{E} [\log P_{\Phi} (Y|G=G_{S}, X =X_{S})].\end{align*}

Due to the lack of diversity in our graph among the samples and the relative sparsity of edges, we consider the nodes, which represent multiple omics datasets, as the primary target for explanation. Specifically, we constructed a mask matrix $M \in R^{n}$ for the nodes. The conditional entropy H can be optimized by adjusting the mask matrix M, $V \odot \sigma (M)$, where $\sigma (M)$ is a node-wise continuous mask for the nodes V, $\sigma $ is the sigmoid function that maps the mask matrix $M$ to $[0,1]^{n}$. Therefore, the optimization objective can be rewritten as follows: 


(6) \begin{align*} \mathop{\min}_{M} -\mathbb{E}[\log P_{\Phi} (Y=y|G=(V \odot \sigma(M), E)].\end{align*}

During the explaining process, the mask matrix $M$ is optimized through gradient descent. In each iteration, the mask matrix $M$ is optimized using the following loss function: 


(7) \begin{align*} Loss_{exp} =&\ \alpha * \log(\hat{y_{n}})\nonumber \\ &+ \beta * \Vert \sigma(M) \Vert_{1}\nonumber \\ &+ \gamma * \sum_{ij} (\sigma(M)_{ij} \log(\sigma(M)_{ij})\nonumber \\ &+ \gamma * \sum_{ij} (1-\sigma(M)_{ij}) \log(1-\sigma(M)_{ij})),\end{align*}

where $\alpha $, $\beta $ and $\gamma $ are three coefficients, $n$ represents the sample belonging to class n, $\hat{y_{n}}$ is the model’s estimated value for the sample in class $n$ and $\Vert \cdot \Vert _{1}$ represents the L1 norm. The first term minimizes the error between the predicted results and the labels. The second term reduces the number of retained nodes, keeping only a small number of important nodes. The third and fourth terms are entropy losses that encourage discrete node feature masks.

Ultimately, the value corresponding to each node in the $\sigma (M)$ matrix represents the importance of that node. We computed the average z-scored importance of each gene across all samples as the final gene importance score. This allows us to select the key genes based on their importance.

### Pathway explanation

The Integrated Gradients method [31] is used to identify key gene pathways. We calculated the IGscore for each feature of each pathway separately using the following formula: 


(8) \begin{align*} IG_{i} = (S_{i} - S_{i}^{\prime}) \int_{0}^{1} \frac{\partial f(S_{i}^{\prime}+\alpha(S_{i}-S_{i}^{\prime})))}{\partial S_{i}^{\prime}}d\alpha.\end{align*}

Here, $S_{i}$ represents the pathway features output from the compressed matrix and $S^{\prime}_{i}$ represents the baseline. The subscripts $i$, respectively, represent the indices of the $i$th feature. $f(\cdot )$ represents the neural network after the compression matrix. Since the features of each pathway are represented by multiple multidimensional features, we took the average of the absolute values of the IGscore for each feature as the IGscore of the pathway. We used the z-score of pathways as the importance scores.

## RESULT

### Datasets

We utilized multi-omics data from The Cancer Genome Atlas (TCGA) database [36] to evaluate our model’s generalizability across three cancer types: Glioblastoma Multiforme (GBM), Lower Grade Glioma (LGG) and Kidney Renal Clear Cell Carcinoma (KIRC). For these cancers, we obtained CNV, mRNA and methylation data from TCGA as model inputs. Additionally, age, a significant factor influencing patient survival time, was included as input for GBM and LGG samples.

The regulatory network among mRNA in the guidance graph was constructed using SCENIC [23], which integrates expression data and TF motif information for more accurate inference of gene regulatory relationships. This network enhances the neural network’s capacity to predict disease risk by providing a precise understanding of cellular functions and signal transduction.

Gene pathways, another important biological information, were utilized in our survival analysis models. We obtained 146 gene pathways from the KEGG database, excluding those specific to certain diseases. These pathways encompass 4989 genes, although not every gene has complete data for all three omics types in the TCGA dataset. Therefore, the actual number of genes used in each omics type is slightly less than 4989, with the specific genes utilized listed in Table 1.

**Table 1:** Benchmark test results

	Our	PathCNN [17]	Linear Regression	Neural Network	SVM	MiNet [34]
GBM	**0.772$\pm $0.006**	0.755$\pm $0.009	0.668$\pm $0.039	0.692$\pm $0.030	0.685$\pm $0.037	0.690$\pm $0.032
LGG	**0.885$\pm $0.006**	0.877$\pm $0.007	0.816$\pm $0.036	0.791$\pm $0.031	0.884$\pm $0.017	0.854$\pm $0.027
KIRC	**0.723$\pm $0.009**	0.709$\pm $0.009	0.654$\pm $0.034	0.702$\pm $0.028	0.684$\pm $0.027	0.659$\pm $0.030

Note: The best results from each dataset are indicated in bold.

Considering the higher fatality rate of GBM, patients with a survival time exceeding 2 years were classified as long-term survivors (LTSs), while those with a survival time less than 2 years were classified as non-LTSs. For LGG and KIRC, which have lower fatality rates compared with GBM, patients with a survival time exceeding 3 years were considered LTSs, while those with a survival time less than 3 years were considered non-LTSs. The LTS and non-LTS groups had 55 and 234 cases for GBM, 156 and 75 cases for LGG and 154 and 69 cases for KIRC, respectively.

### Feature validity analysis and visualization

**Figure 3:**
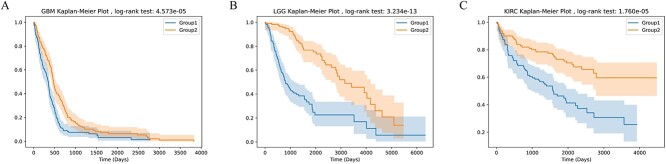
The patients were divided into high-risk and low-risk groups based on the predicted median, and there was a significant difference between the two groups in Kaplan–Meier analysis.

**Figure 4:**
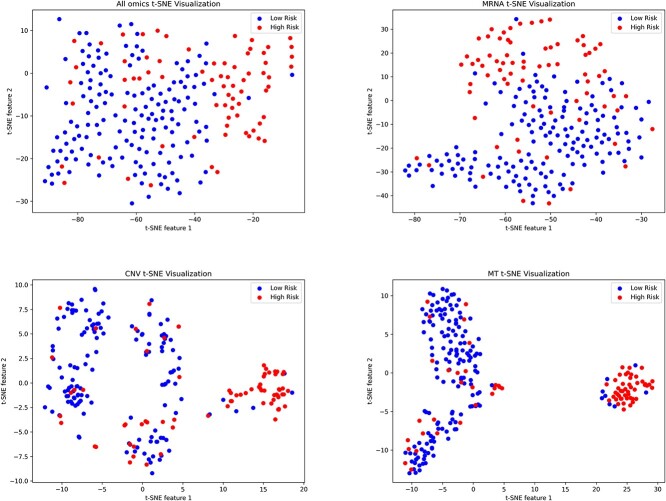
t-SNE was used to visualize the features of pathways trained on the LGG model. It can be observed that in all three omics and overall, there is a clear distinction in pathway features between high-risk and low-risk patients.

Using the LGG dataset as an example, we initially conducted tests on this dataset. Based on the model’s output, patients were classified into two subtypes: high-risk and low-risk. The Kaplan–Meier curve visualized a significant difference in survival time between these two subgroups (Figure 3). Subsequently, pathway-level features were separately visualized for each omics and overall using t-SNE (Figure 4). t-SNE effectively compressed the features into a 2D space, revealing distinct separability of samples from the two subtypes based on pathway-level features. This implies that by employing graph neural networks and pathway aggregation, discriminative pathway features can be extracted, thereby enabling subsequent neural networks to more effectively predict patient risk.

### Benchmark comparision

We compared our approach with two state-of-the-art models, PathCNN [17] and MiNet [34], as well as three classical methods, Linear Regression, Neural Network and Support Vector Machine (SVM), across four cancer datasets. We conducted 30 experiments using 5-fold cross-validation on each dataset, without a specifically partitioned test set. Similar to PathCNN, we combined all test sets from the 5-fold cross-validation, which encompasses the entire dataset, to calculate the Area Under Curve (AUC). The evaluation metric used in each experiment was the AUC of the entire dataset. The average AUC from the 30 experiments served as a comparative measure to assess the performance of each model. Table 2 presents the experimental results for all models on each dataset, demonstrating that our approach achieved superior AUC values compared with other methods across all three cancer datasets.

**Table 2:** The results of different omics combinations

	MRNA	MRNA&CNV	MRNA&MT	MRNA&CNV&MT
GBM	0.752$\pm $0.0122	0.765$\pm $0.0070	0.765$\pm $0.0059	0.772$\pm $0.0060
LGG	0.847$\pm $0.0156	0.871$\pm $0.0078	0.868$\pm $0.0064	0.885$\pm $0.0064
KIRC	0.710$\pm $0.0118	0.720$\pm $0.0085	0.708$\pm $0.0080	0.723$\pm $0.0091

### Ablation study

This section presents some ablation experiments for this study. First, we conducted ablation experiments on the main method using the LGG dataset (Table 3). To evaluate the effectiveness of pathway aggregation, we used a Neural Network as the baseline for comparison. We then experimented with a network that only contains the pathway aggregation module and the prediction head. This network achieves better AUC and stability compared with neural networks without pathway aggregation. By comparing the results of using a linear layer and a graph neural network before the aggregation module, it is evident that the graph neural network module can further enhance performance. In addition, to demonstrate the superior capability of the aggregation module, we replaced it with other dimensionality reduction methods for comparison. Since our dimensionality reduction module is used in neural networks, we selected two of the most commonly used dimensionality reduction modules in neural networks: max pooling and average pooling. For traditional dimensionality reduction methods, we chose an unsupervised dimensionality reduction method, PCA, for comparison. The results are presented in Table 4. As can be seen from the table, our method exhibits better performance. Although the 2/3-year division is the main division method in previous research [17, 37], we attempted experiments with different time divisions, and the results are shown in Supplementary Table 1 (see Supplementary Data available online at http://bib.oxfordjournals.org/). Finally, we attempted to use other clinical information in the model, and the results are presented in Supplementary Table 3 (see Supplementary Data available online at http://bib.oxfordjournals.org/). The results show that our method can achieve better results under most time divisions and other clinical data.

**Table 3:** Method ablation experiments

Pathway Aggergation	Linear	GNN	AUC
			0.791$\pm $0.031
✓			0.836$\pm $0.009
✓	✓		0.879$\pm $0.006
✓		✓	0.885$\pm $0.006

**Table 4:** Ablation study for reduction methods

	Our	MaxPooling	AvgPooling	PCA
GBM	0.772$\pm $0.006	0.723$\pm $0.013	0.713$\pm $0.005	0.733$\pm $0.006
LGG	0.885$\pm $0.006	0.811$\pm $0.017	0.855$\pm $0.011	0.830$\pm $0.014
KIRC	0.723$\pm $0.009	0.609$\pm $0.014	0.634$\pm $0.022	0.670$\pm $0.022

### Multi-omics data improve model performance

To demonstrate the model’s enhanced performance using multi-omics data and identify comparatively important omics, we conducted tests with different combinations of omics. Since mRNA data serve as a central bridge in the model, it was retained in all combinations. In two-omics combinations, edges between mRNA nodes in the guidance graph and between the two omics were preserved. Combinations having been tested separately include only mRNA, mRNA and CNV, and mRNA and MT, as illustrated in Table 5.

**Table 5:** The gene explain results

Gene	Omics	Importance Score	$P$ -value	Adjusted $P$-value
Sec61g	mRNA	17.39	1.567e-30	3.908e-27
Cyp27b1	mRNA	16.72	2.638e-31	1.315e-27
Cdk4	mRNA	8.311	2.074e-09	1.724e-07
Ifna1	CNV	8.560	2.437e-13	5.066e-11
Ifna6	CNV	8.308	3.156e-12	4.498e-10
Ifna8	CNV	8.302	1.723e-13	4.093e-11
Plcg1	MT	26.49	2.987e-04	0.01795
Hexb	MT	14.67	6.243e-04	0.03244
Prkaca	MT	12.51	9.378e-11	9.357e-08

From the table, it can be observed that using only mRNA data gets the worst performance. The performance is similar when using two omics combinations, while it is best when utilizing data from all three omics simultaneously. This demonstrates that increasing the number of omics does improve the model’s performance, highlighting each individual omic’s importance. Furthermore, experiments using the combination of mRNA and CNV sometimes yield higher AUC means compared with those obtained with the combination of mRNA and MT. However, considering standard deviation values, results from combining mRNA and MT are more stable than those from combining mRNA and CNV. This suggests that, in survival prediction, CNV contains more potentially useful information than MT, but the model also finds it more challenging to effectively utilize the information from CNV.

### Identification of key genes and pathways

**Figure 5:**
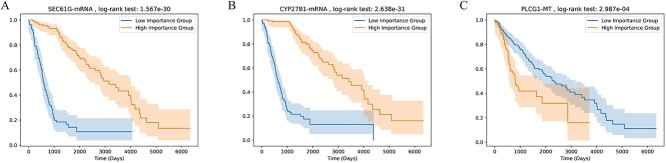
Kaplan–Meier curves for genes, dichotomized into two groups based on the median split of gene importance scores. The shaded area represents the 95% confidence interval.

We identified key genes using the GNN-Explainer-based interpretation method, and the results for LGG are shown in Table 6. The mean of the gene’s importance scores across all samples is taken as the gene’s overall importance score. For each omics, we selected the top three important key genes with adjusted $P$-values less than 0.05 for display. We divided patients into two groups, a high-importance-score group and a low-importance-score group, based on the mean importance score of a specific gene. Using this grouping, we conducted survival analysis on the two groups of patients, as shown in Figure 5. In previous studies, these genes were also found to be associated with diseases. Zeng *et al.* [38] identified SEC61G as a pivotal regulator that promotes immune evasion and tumor growth in glioblastoma. Zhang *et al.* [39] found that CYP27B1 is overexpressed in LGGs, and its overexpression is associated with poor prognosis. Li *et al.* [40] found a significant correlation between the expression of PLCG1 and IDH1/2 status and patients’ clinical outcomes. Additionally, PLCG1-targeted drugs significantly inhibited tumor growth in IDH wild-type LGG cell lines and in mouse models.

**Table 6:** The number of genes included in each omics

Cancer	mRNA	CNV	MT
GBM	3914	4876	3416
LGG	4631	4842	3675
KIRC	4633	4835	3920

**Figure 6:**
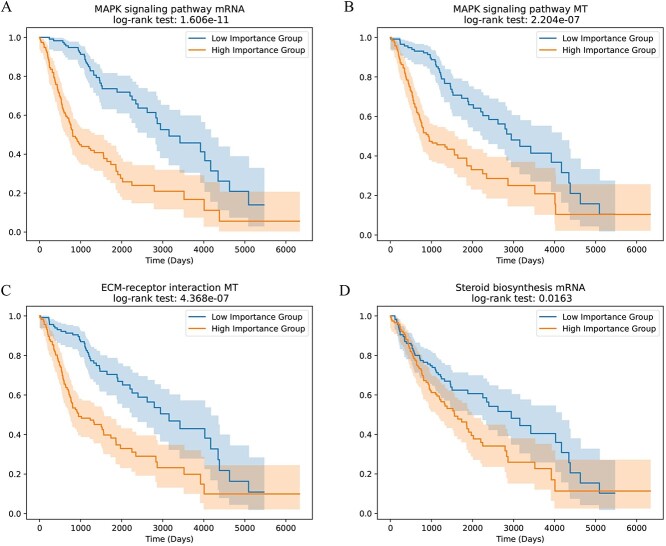
Kaplan–Meier curves for pathway, dichotomized into two groups based on the median split of pathway importance scores. The shaded area represents the 95% confidence interval.

We identified pathways with high importance scores in each omics using the Integrated Gradients method. The importance score for each pathway was determined by taking the median of all samples’ importance scores for that pathway. Pathways with z-score transformed importance scores greater than 1.96 were considered key pathways. In the LGG dataset, we identified a total of 19 key pathways, including 8 key pathways in mRNA omics, 4 key pathways in CNV omics and 7 key pathways in methylation omics. The importance score of a pathway reflects its impact on the prediction outcome. A higher score indicates a greater influence on the result. Therefore, we divided the samples into high and low score groups based on the median importance score of pathways and conducted survival analysis. Among the 19 pathways, 18 showed significant significance ($P$-value < 0.05) in survival analysis. Table 7 shows 18 pathways with significant correlation, along with their importance scores and $P$-values. Figure 6 displays the survival analysis for several pathways. MAPK has been identified as a key pathway in mRNA and MT omics. This is further supported by the survival analysis using importance scores, showing a strong correlation. Additionally, in previous studies, Nageswara *et al.* [41] identified the key regulatory factor of the MAPK signaling pathway in the occurrence of pediatric LGG tumors. Apart from this, other pathways have also been mentioned in previous studies. Jiang *et al.* [42] found that overexpressed crosstalk genes may be involved in the progression and poor prognosis of LGG through the ECM-receptor interaction pathway. Hirtz *et al.* [43] demonstrated the association between the expression levels of steroid biosynthesis enzymes and the survival risk of LGG patients.

**Table 7:** The pathway explain results

Pathway	Omics	Importance Score	$P$ -value
Tgf beta signaling pathway	mRNA	4.962	0.00142
Steroid biosynthesis	mRNA	3.774	0.0163
MAPK signaling pathway	mRNA	3.655	1.606e−11
Ribosome	mRNA	2.8	0.0153
Glutathione metabolism	mRNA	2.675	0.013
Endocytosis	mRNA	2.337	1.823e−06
Erbb signaling pathway	mRNA	2.26	7.447e−06
Protein export	mRNA	1.983	0.00133
Cytosolic dna sensing pathway	CNV	5.99	5.567e−08
Cytokine cytokine receptor interaction	CNV	4.654	2.614e−09
Rig i like receptor signaling pathway	CNV	4.085	5.544e−07
Natural killer cell mediated cytotoxicity	CNV	3.569	6.175e−12
Regulation of actin cytoskeleton	MT	4.609	4.019e−08
Neuroactive ligand receptor interaction	MT	4.026	3.977e−07
MAPK signaling pathway	MT	3.838	2.204e−07
Endocytosis	MT	3.601	8.831e−04
Lysosome	MT	3.405	9.078e−07
ECM-receptor interaction	MT	2.931	4.368e−07

Furthermore, we identified important pathways in GBM, and the results are presented in Supplementary Table 4 (see Supplementary Data available online at http://bib.oxfordjournals.org/). It can be seen that we identified some common pathways, such as Cytokine cytokine receptor interaction in mRNA omics and Neuroactive ligand receptor interaction in CNV omics. We also explained some pathways that have been validated in previous studies, such as the JAK-STAT signaling pathway [44] and Focal adhesion [45]. Experiments using different omics have shown that methylation omics is important for our model. Therefore, compared with PathCNN, which did not identify any methylation omics, we identified four important pathways in methylation omics: Neuroactive ligand receptor interaction, Cytokine cytokine receptor interaction, Focal adhesion and Ubiquitin mediated proteolysis.

## CONCLUSION

We have proposed a novel risk assessment algorithm, the multiomics-GNN, which effectively integrates multi-omics data, gene regulatory networks and pathway information to extract features and improve accuracy in predicting survival risk. Our model outperforms conventional methods when applied to diverse cancer datasets with multi-omics data. Moreover, we have demonstrated that our method based on multiple omics data achieves superior performance than on single omics data. Each additional omics contributes significantly and essentially to enhancing predictive performance, highlighting the effectiveness of the correlation-based multi-omics guidance graph we constructed. Furthermore, employing two interpretation methods at both the gene and pathway levels has revealed key factors that are strongly correlated with risk in survival analysis, and many of which have been corroborated by previous studies. In summary, this study showcases the potential of comprehensively and hierarchically incorporating multi-level information including multi-omics data, pathway information and gene regulatory information with graph neural network for accurate risk prediction while identifying nonlinear and risk-associated key factors. Moreover, graph neural networks demonstrate significant potential in processing multi-omics data and regulatory networks. Employing graph neural networks for handling and integrating data at various levels can enhance disease analysis, thereby facilitating the future integration of more omics data or the incorporation of MRI imaging data.

**Key Points**

- We proposed a Multilevel Graph Neural Network (Multilevel-GNN) algorithm, which hierarchically combines multi-omics data, gene regulatory networks and pathway information and improved the prediction performance compared with the existing methods.
- Multilevel-GNN constructed a guidance graph by explicitly establishing associations between and within omics data and employed a graph neural network to extract gene features that entail interactions between genes based on the guidance graph.
- Multilevel-GNN employed a learnable linear layer, which aggregates gene-level features into pathway-level features representing specific meaningful biological functions, thereby enhancing the model’s accuracy as well as enabling interpretation at the pathway level.
- Multilevel-GNN employed two interpretation algorithms, GNN-Explainer and IGscore, to non-linearly identify key factors at the gene and pathway levels, enhancing the interpretability of neural network prediction decisions.

## Supplementary Material

Supplimentary_Meterial_1_bbae184

## Data Availability

Our program is in https://github.com/Y-Claw/Multilevel-GNN. All the data comes from public databases, which can be obtained from public databases or from our GitHub projects.

## Author Biographies

**Hongxi Yan** is a phd candidate student at Beihang University.

**Dawei Weng** is a lecturer at Capital Medical University.

**Dongguo Li** is an associate professor at Capital Medical University.

**Yu Gu** is a professor at Capital Medical University.

**Wenji Ma** is a professor at Shanghai Jiao Tong University School of Medicine.

**Qingjie Liu** is an associate professor at Beihang University.
