# Supplementary material for: Prior knowledge-guided multilevel graph neural network for tumor risk prediction and interpretation via multi-omics data integration
Source: Brief Bioinform. 2024 Apr 25;25(3):bbae184. doi: 10.1093/bib/bbae184 (PMC11052635; doi:10.1093/bib/bbae184)
Supplement: Supplimentary_Meterial_1_bbae184 [file supplimentary_meterial_1_bbae184.pdf]

Supplimentary Meterial

April 20, 2024

1 Experiments with Different Time Divisions

In this section, we classified patients with LGG into high-risk and low-risk groups according to different time points and conducted comparative experiments with deep learning-based methods. The results are presented in the Table 1.

	Our	Pathcnn	MiNet	Neural Network
2 year	0.847±0.012	0.857±0.008	0.844±0.034	0.808±0.022
2.5 year	0.913±0.009	0.877±0.006	0.878±0.015	0.855±0.017
3 year	0.885±0.006	0.877±0.007	0.854±0.027	0.791±0.031
3.5 year	0.826±0.008	0.806±0.006	0.816±0.041	0.809±0.023
4 year	0.794±0.012	0.792±0.007	0.740±0.028	0.760±0.027

Table 1: Experiments with Different Time Divisions

2 Experiments with Different Clinical Information

	Age	Sex	Region
GBM	3.06e-09	7.21e-04	0.035
LGG	2.63e-16	1.00	0.199

Table 2: Clinical Information Pvalue

We calculated the p-values for the association between gender and location with patient risk in LGG and GBM. The results (Table 2) showed that there was no significant correlation between clinical information and patient risk in LGG. In GBM, other clinical information had a higher correlation with the true values, so we conducted further testing on the GBM dataset. Since gender and Region are both discrete variables, we map them into a embedding and input

	Our	Pathcnn	MiNet	Neural Network
None	0.672±0.011	0.669±0.014	0.665±0.023	0.670±0.014
Age	0.772±0.006	0.755±0.009	0.690±0.032	0.692±0.030
Sex	0.705±0.010	0.676±0.004	0.621±0.029	0.683±0.021
Region	0.686±0.012	0.672±0.009	0.647±0.037	0.675±0.024
ALL	0.780±0.007	0.762±0.011	0.714±0.025	0.706±0.025

Table 3: Experiments with Different Clinical Information

them into the network. The results are shown in Table 3. The results show that using more case features in GBM does improve the performance of our model.

3 GBM Pathway Explain Result

We conducted an interpretation of important pathways in GBM to compare the results of PathCNN. The results are shown in Table 4

Pathway	Omics	Importance Score	P-value
Focal adhesion	mRNA	5.126	2.744e-05
JAK-STAT signaling pathway	mRNA	4.440	6.604e-07
Antigen processing and presentation	mRNA	4.036	0.0476
Cytokine cytokine receptor interaction	mRNA	3.976	9.771e-04
MAPK signaling pathway	mRNA	3.663	0.714
Complement and coagulation cascades	mRNA	3.190	0.0113
Neuroactive ligand receptor interaction	CNV	9.072	0.00283
MAPK signaling pathway	CNV	4.048	8.330e-06
Olfactory transduction	CNV	3.894	4.357e-07
Regulation of actin cytoskeleton	CNV	2.415	3.058e-04
Neuroactive ligand receptor interaction	MT	7.493	6.813e-15
Cytokine cytokine receptor interaction	MT	4.676	4.643e-06
Focal adhesion	MT	3.433	2.710e-08
Ubiquitin mediated proteolysis	MT	2.533	6.065e-12

Table 4: GBM Pathway Explain Result
